# Understanding Systemic and Local Inflammation Induced by Nasal Polyposis: Role of the Allergic Phenotype

**DOI:** 10.3389/fmolb.2021.662792

**Published:** 2021-05-14

**Authors:** María Isabel Delgado-Dolset, David Obeso, Javier Sánchez-Solares, Leticia Mera-Berriatua, Paloma Fernández, Coral Barbas, Miguel Fresnillo, Tomás Chivato, Domingo Barber, María M. Escribese, Alma Villaseñor

**Affiliations:** ^1^ Department of Basic Medical Sciences, Faculty of Medicine, Institute of Applied Molecular Medicine (IMMA), San Pablo CEU Universities, Madrid, Spain; ^2^ Centre for Metabolomics and Bioanalysis (CEMBIO), Department of Chemistry and Biochemistry, Faculty of Pharmacy, San Pablo CEU Universities, Madrid, Spain; ^3^ Otorhinolaringology Service, HM Montepríncipe Hospital, Madrid, Spain; ^4^ Department of Clinic Medical Sciences, Faculty of Medicine, San Pablo CEU Universities, Madrid, Spain; ^5^ Department of Basic Medical Sciences, Faculty of Medicine, San Pablo CEU Universities, Madrid, Spain

**Keywords:** metabolomics, nasal polyps, allergy, targeted analysis, untargeted analysis

## Abstract

Chronic rhinosinusitis with nasal polyps (CRSwNP) is characterized by persistent symptoms associated to the development of nasal polyps. To this day, the molecular mechanisms involved are still not well defined. However, it has been suggested that a sustained inflammation as allergy is involved in its onset. In this exploratory study, the aim was to investigate the effect of the allergic status in the development of CRSwNP. To achieve this, we recruited 22 patients with CRSwNP and classified them in non-allergic and allergic using ImmunoCAP ISAC molecular diagnosis. Plasma samples were analyzed using liquid chromatography coupled to mass spectrometry (LC-MS). Subsequently, significant metabolites from plasma that were commercially available were then analyzed by targeted analysis in some nasal polyps. Additionally, nasal polyp and nasal mucosa samples were examined for eosinophils, neutrophils, CD3^+^ and CD11c^+^ cells, as well as collagen deposition and goblet cell hyperplasia. We found that 9 out of the 22 patients were sensitized to some aeroallergens (named as allergic CRSwNP). The other 13 patients had no sensitizations (non-allergic CRSwNP). Regarding metabolomics, bilirubin, cortisol, lysophosphatidylcholines (LPCs) 16:0, 18:0 and 20:4 and lysophosphatidylinositol (LPI) 20:4, which are usually related to a sustained allergic inflammation, were unexpectedly increased in plasma of non-allergic CRSwNP compared to allergic CRSwNP. LPC 16:0, LPC 18:0 and LPI 20:4 followed the same trend in nasal polyp as they did in plasma. Comparison of nasal polyps with nasal mucosa showed a significant increase in eosinophils (*p* < 0.001) and neutrophils (*p* < 0.01) in allergic CRSwNP. There were more eosinophils in polyps of non-allergic CRSwNP than in their nasal mucosa (*p* < 0.01). Polyps from non-allergic CRSwNP had less eosinophils than the polyps of allergic CRSwNP (*p* < 0.05) and reduced amounts of collagen compared to their nasal mucosa (*p* < 0.001). Our data suggests that there is a systemic inflammatory response associated to CRSwNP in the absence of allergy, which could be accountable for the nasal polyp development. Allergic CRSwNP presented a higher number of eosinophils in nasal polyps, suggesting that eosinophilia might be connected to the development of nasal polyps in this phenotype.

## Introduction

Chronic rhinosinusitis with nasal polyps (CRSwNP) is a disease characterized by persistent inflammatory symptoms in nasal and paranasal mucosa that result in the development of a nasal polyp (Fokkens et al., 2012). This is an outgrowth of tissue that arises into the nasal cavity (Schleimer, 2017). The prevalence of the disease is estimated between 1–4% of the population (Fokkens et al., 2012; Chen et al., 2020). Symptoms include nasal blockage and itching, rhinorrhea, sneezing, facial pain, headache and smell impairment or loss (Georgy and Peters, 2012; Chen et al., 2020). Treatment starts with intranasal topic corticosteroids in the milder cases, followed by surgical extirpation or biological drugs, such as omalizumab, in the most severe ones (Lund, 1995; Georgy and Peters, 2012).

CRSwNP presents several endotypes, which are subtypes of diseases defined either by having the same molecular mechanism or because they respond to the same treatment (Anderson, 2008). In this sense, the analysis of cells and molecules involved in inflammation and the underlying pathophysiological mechanism is essential in CRSwNP (Brescia et al., 2020).

There are several comorbidities associated to CRSwNP appearance and recurrence, including asthma, allergic rhinitis, cystic fibrosis or aspirin-exacerbated respiratory disease (AERD) (Lund, 1995; Wynn and Har-El, 2004; Pearlman et al., 2010). In addition, nasal polyps relapse in up to 60–70% of the patients (Wynn and Har-El, 2004).

There are significant differences in histological features between nasal mucosa and nasal polyps, such as the development of oedema or fibrosis, goblet cell hyperplasia and/or squamous metaplasia; the thickening of the basal membrane; and the infiltration of inflammatory cells, including lymphocytes, eosinophils and neutrophils (Ferreira Couto et al., 2008).

However, the molecular mechanisms involved in the development of nasal polyps are still not well defined (Hopkins, 2019). Due to the inflammatory features described in several cohorts of CRSwNP (Brescia et al., 2018), most of the hypothesis agree that long-term maintained inflammation plays a key role in this process (Tos et al., 2010; Chojnowska et al., 2013; Brescia et al., 2021) and, therefore, inflammatory diseases such as allergy and/or asthma might be associated with its onset.

The role of allergy in CRSwNP development has been extensively discussed. In their 2014 review, Wilson *et al* (Wilson et al., 2014) examined the existing evidence both for and against an association of these two diseases. Although the role of allergy in CRSwNP was inconclusive, the review showed a higher rate of positive skin prick test among CRSwNP patients; and greater improvement in patients with negative skin prick tests compared to those with positive ones. These differences suggest that allergic inflammation might play a role in nasal polyposis.

Metabolomics is one of the most promising tools in the identification of biomarkers. It allows the detection of dynamic changes and alterations in the metabolism that point to a given pathological state (Villaseñor et al., 2017). The metabolome is closely linked to the phenotype and can be an extremely useful tool for diagnosing diseases and evaluating the effect of treatments. From a practical point of view, it uses very sensitive and specific techniques, such as liquid chromatography coupled to mass spectrometry (LC-MS), which allows the simultaneous detection of a great variety of metabolites in a biological sample (Crestani et al., 2020). Once the study subjects are well characterized, these results should be validated in a new and larger cohort of patients in subsequent studies. Compared to other omics, such as transcriptomics or genomics, the validation of metabolites found after exploratory studies is carried out through the development of analytical methodologies. This process is usually laborious depending on the number of metabolites and their physicochemical properties, and is conditioned by the availability of commercial standards.

Previous reports show that allergic inflammation has both systemic and local effects. As for the systemic role of allergy, we have previously demonstrated that severe allergic phenotypes induce significant changes in the plasma metabolome (Obeso et al., 2018). Similarly, allergic inflammation also induces local remodeling in oral mucosa (Aceves and Ackerman, 2009; Samitas et al., 2018; Rosace et al., 2019). In fact, this remodeling might result in the formation of the nasal polyp (Tos et al., 2010). However, to our knowledge, no metabolomic analysis has been performed for CRSwNP.

Here, our aim is to determine the role of allergic inflammation in patients with CRSwNP and how it affects both systemic and local features. Thus, we performed: 1) a systemic analysis, using a non-targeted metabolomics approach to achieve an overall picture of the metabolic profile in CRSwNP patients with and without allergy, and 2) a local analysis using both a targeted metabolomic analysis of the nasal polyps to evaluate if the systemic potential metabolites found are associated specifically to the nasal polyp, and a histological analysis to better characterize the local polyp environment. Our results support the idea that the nasal polyp has a specific inflammatory environment characterized by immune cells infiltrates, epithelial damage and the presence of inflammatory-related metabolites.

## Materials and Methods

### Patients and Sample Collection

Twenty-two adult patients diagnosed with CRSwNP within an age range of 48 ± 8 years that arrived for the first time to the Otorhinolaryngology Service of the Hospital Madrid Monteprincipe (Spain) were included in this study. Patients underwent a basic allergy history questionnaire. The inclusion criteria were: patients older than 18 years of age, with nasal polyps that needed to undergo a surgery to remove following their clinician’s diagnosis. Patients received the same pharmacological pre-surgery treatment and their corticosteroid medication was removed two weeks prior the procedure. The exclusion criteria were: patients with concomitant inflammatory diseases such as autoimmune diseases or cancer. The ethical committee approved the study protocol and all subjects were informed and provided written consent prior to any procedure. Data were anonymized.

During endoscopic surgical procedures to remove the polyp, 5 mm biopsies of nasal polyp and nasal mucosa were obtained and either kept in RNAlater at −80°C or fixed in 4% paraformaldehyde (PFA) for 24–48 h. PFA-fixed samples were dehydrated and included in paraffin using Leica TP 1020 tissue processor. From these, 3 μm sections were obtained and used for histological and immunohistochemical analysis. Additionally, from the twenty-two patients, a blood sample from nineteen was obtained. About 20 ml of heparinized blood were collected to obtain plasma using a Ficoll-Paque (GE Healthcare™) density gradient centrifugation. Supernatants were stored at −80°C until their use.

### ImmunoCAP ISAC

To determine the sensitization profile of the patients, ImmunoCAP ISAC® (Phadia, Uppsala, Sweden) with chips for 112 allergens assays were performed to detect specific IgE (sIgE) as described in the manufacturer protocol. Values above 0.3 ISU-E were considered positive.

### Untargeted Plasma Metabolomic Analysis

Plasma samples were measured using a Liquid Chromatography coupled to Mass Spectrometry (LC-QTOF-MS Agilent series 6520). We followed previously described methodologies developed in our group (Ciborowski et al., 2010). Principal component analysis and heatmaps with hierarchical clustering were obtained using MetaboAnalyst v 5.0 webpage (https://www.metaboanalyst.ca).

Full descriptions are available in the Supplementary Information. Metabolite annotation was carried out using the online advanced CEU Mass Mediator tool (Godzien et al., 2015; Dudzik et al., 2018; Gil-de-la-Fuente et al., 2019). Statistical analysis was performed using non-parametric Mann Whitney *U* test, with statistical significance set at 95% (*p* < 0.05) with a Benjamini-Hochberg (also known as False Discovery Rate, FDR). Annotation was confirmed through LC-MS/MS experiments using 20 eV for fragmentation. Data were uploaded to Metabolomics Workbench webpage (ID study: ST001733 and ST001734).

### Target Method for Nasal Polyps

#### Sample Preparation

Three nasal polyp samples of each group collected in RNAlater were used to measure the metabolites from plasma that were commercially available. RNAlater solvent was removed by washing the tissue 3 times with PBS 1X. Then, the nasal polyp was frozen in liquid nitrogen for 30 s. The frozen sample was put in cryoPREPTMCP02 (Covaris, MA, United States) plastic bags and submerged again for 30 s in liquid nitrogen. Once the plastic bag was inside the automated cryoPREPTMCP02, two consecutive impact forces of levels 2 and 4 out of 6 were applied. The resultant powder was gathered and weighted. Then, 100 µL of cold methanol:ethanol (1:1, v/v) and 0.5 µL of internal standard (LPC 18:1-d7; 0.01 mM) were added per each 10 mg of tissue for metabolite extraction and protein precipitation. Samples were then vortex-mixed and homogenized using Tissue-Lyser LT homogenizer (Qiagen, Germany) for 5 min at 50 Hz, 3 times. Supernatant containing the metabolites was separated from the pellet by centrifugation (2,000 rcf for 10 min at 4°C). Then, an aliquot of 70 µL was transferred to an LC vial and diluted with 490 µL of mobile phase (5% water: 95% acetonitrile; both with 7.5 mM ammonium acetate and 0.1% acetic acid). All samples were randomized before metabolite extraction and for the corresponding analytical run.

#### Analysis of Nasal Polyp by Liquid Chromatography Coupled to Triple Quadrupole Mass Spectrometry (LC-QqQ-MS)

Samples were measured using Dynamic Molecular Reaction Monitoring (dMRM) in LC-QqQ-MS for the analysis of metabolites. We used HPLC system (1260 Infinity, Agilent Technologies) coupled to an ESI(AJS)-QqQ-MS (6470 Agilent Technologies). The metabolites were separated on a Kinetex hydrophilic interaction liquid chromatography (HILIC) silica column (150 mm × 2.1 mm, particle size 100Å, Phenomenex, United States) maintained at 25°C. The mobile phases consisted of: A) water, and B) acetonitrile, both with 7.5 mM ammonium acetate and 0.1% acetic acid, with a final pH of 4.0 in the aqueous phase. The flow rate was 0.5 ml/min with an injection volume of 5 µL. Gradient started with 5% of A for 2 min, then increased up to 50% until 12 min, and back to initial conditions until 22 min. The MS conditions were: 5500 V of capillary voltage in positive ESI mode, a nebulizer gas flow rate of 11.0 L/min; a source temperature of 250°C; and a source pressure of 60 psi. The sample tray temperature was maintained at 4°C. Each transition was optimized adjusting the fragmentor and collision energy voltages, which can be seen in Table 1.

**TABLE 1:** Optimized MS transitions and parameters for the targeted analysis. LPC: Lysophosphatidylcholine, LPI: Lysophosphatidylinositol, RT: Retention time, V: Volt, eV: Electronvolt.

Compound name	Precursor ion (*m/z*)	Product ion (*m/z*)	RT (min)	Fragmentor (V)	Collision energy (eV)	Polarity
Bilirubin	585.3	299.1	0.91	131	25	+
LPC 16:0	496.3	183.9	9.95	100	28	+
LPC 18:0	524.4	183.8	9.80	100	28	+
LPI 20:4	621.3	361.3	7.83	84	17	+

#### Data Acquirement, Pre-processing and Treatment

Data were acquired in MassHunter Workstation B.05.00 (Agilent Technologies), and re-processed using MassHunter QQQ Quantitative Analysis B.08.00 (Agilent) where peak areas were integrated. Concentration of metabolites were calculated using external calibration curves with the standard addition method. Once the concentrations were obtained, all data treatment and statistical comparisons was performed using Excel 2016, MATLAB R 2018b and GraphPad Prism v8.1.2.

### Immune Cell Quantification

#### Tissue Staining

We quantified eosinophils, neutrophils, CD3^+^ and CD11c^+^ cells, and checked for collagen deposition and goblet cell hyperplasia, in nasal polyps and nasal mucosa samples. For eosinophil quantification, we adapted a Luna staining protocol. Samples were stained with a working solution prepared with 0.9 parts of homemade Weigert’s Iron Hematoxylin, and 0.1 parts of commercial Briebrich-Scarlett solution (Sigma Aldrich, ref. HT151) for 5 min. Slides were then differentiated in 1% acid alcohol and 0.25% lithium carbonate solutions, and preparations were mounted.

Regarding goblet cell hyperplasia, we optimized a Periodic Acid-Schiff (PAS) staining. Briefly, samples were kept in a 0.5% periodic acid solution, then stained with Schiff’s reactive (Merck, ref. 109033) and washed. Nuclei were stained with a 1:4 Harris Hematoxylin solution and differentiated with a 1% acid alcohol (1% of HCl in 70% ethanol) solution and a saturated lithium carbonate (Sigma-Aldrich, ref. 62470) solution. Preparations were mounted with DPX medium.

Regarding neutrophil, CD3^+^ cells and CD11c^+^ cells quantification, we performed immunohistochemical staining with anti-human neutrophil elastase (ab68672, ABCAM), anti-CD3 (MCA 1477, AbD Serotec) and anti-CD11c (NCL-L-CD11c-563, Novocastra) using the automatized system Leica Bond Max (Leica Biosystems), as previously described (Sanchez-Solares et al., 2019). For negative controls, the antibody was substituted with antibody diluent Bond™ (Leica Biosystems) for incubation.

Masson Trichrome staining (Sigma-Aldrich, ref HT15-1KT) was performed following the manufacturer’s instructions.

#### Image Analysis

All slides were scanned with Leica SCN400 scanner (Leica Biosystems). Images were obtained for each staining using the Leica Scan Viewer software. Luna and neutrophil elastase-positive cells were counted in the whole sample, while CD3^+^ cells and CD11c^+^ cells were counted in five representative areas on the sample. Areas were measured using ImageJ v1.51j8 by at least two independent observers. Results are presented as number of cells per area. For goblet cell hyperplasia, PAS^+^ stained areas in the epithelium were measured, while for collagen deposition, green areas of Masson staining were measured in the whole sample using Image-Pro Plus v4.5.0.29 for Windows (Media Cybernetics) software.

#### Statistical Analysis

GraphPad Prism v8.1.2 for Windows (GraphPad Software) was used for statistical analysis. We checked data distribution and then used *t*-test or Mann-Whitney *U* test to determine significant differences between the means accordingly. Statistical significance was set at 95% (*p* < 0.05).

## Results

### Patient Classification

**FIGURE 1:**
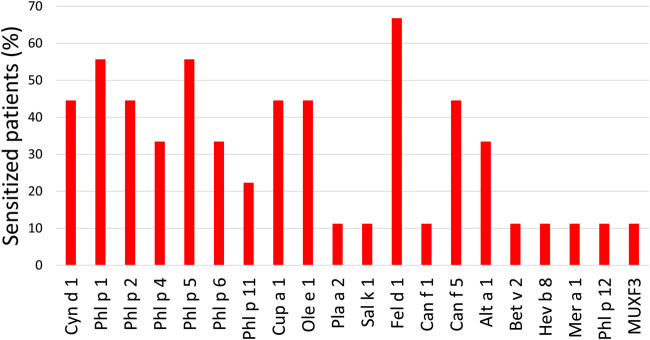
Percentage of allergic patients sensitized to each allergen. Cyn d: *Cynodon dactylon* (Bermuda grass); Phl p: *Phleum pratense* (timothy); Cup a: *Cupressus arizonica* (cypress); Ole e: *Olea europaea* (olive); Pla a: *Platanus acerifolia* (London plane tree); Sal k: *Salsola kali* (saltwort); Fel d: *Felis domesticus* (cat); Can f: *Canis familiaris* (dog); Alt a: *Alternaria alternata* (*Alternaria* plant rot fungus); Bet v: *Betula verrucose* (European white birch); Hev b: *Hevea brasiliensis* (latex); Mer a: *Mercurialis annua* (annual mercury); MUXF3: Bromelain.

Patients with CRSwNP were classified according to their allergic sensitization phenotype by ImmunoCAP ISAC (Figure 1). As observed in the figure, 13 patients (59.1%) had undetectable sIgE levels and were classified as non-allergic CRSwNP. On the other hand, 9 (40.9%) had significant levels of sIgE. They were sensitized to either one or more perennial allergen sources (*i.e.* cat, dog, mites or *Alternaria*) and/or to a group of seasonal allergen sources. Thus, we consider them to be perennial allergic patients and classified them as allergic CRSwNP patients.

### Untargeted Plasma Metabolomic Analysis

The plasma metabolic profile of each patient with CRSwNP with or without allergic sensitization was acquired. After data treatment, 535 features for LC-MS positive and 429 for LC-MS negative ionization modes in each plasma sample were obtained. These features passed the different quality filters (blank subtraction, presence in quality control samples (QCs; >50%) and patients (>75%), and coefficient of variation in QCs (<30%)).

**FIGURE 2:**
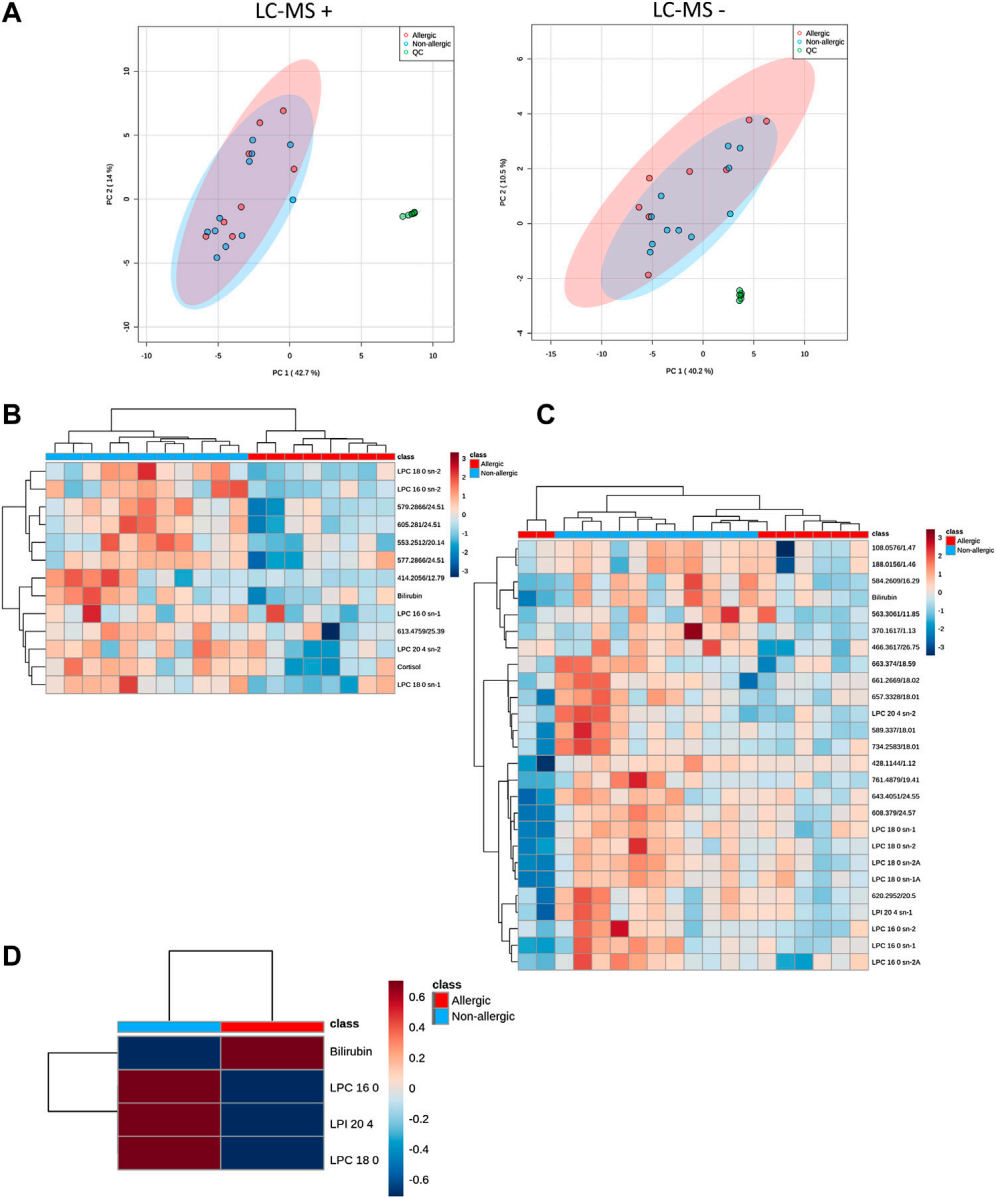
**(A).** Unsupervized PCA for QC injections (●; green dot), allergic patients with CRSwNP (●; red dot) and non-allergic patients with CRSwNP (●; blue dot) models showing the quality of the data for LC-MS. Confidence regions for each group were set at 95% and are depicted with the corresponding color group. Data were centered scaled and Log transformed. **(B,C).** Significant signals were depicted on a heatmap using hierarchical clustering of the samples (represented in columns) and metabolites (in rows) for ESI + and ESI − , respectively. Red cells represent higher levels of the specific metabolite in that sample, whereas blue cells represent lower levels. Samples and metabolites are clustered according to their similarity. Mann-Whitney *U* test with a Benjamini-Hochberg correction was used to detect statistical significance (*p* < 0.05). Unknown features are represented by “Mass@Retention Time.” **(C).** Heatmaps of significant plasma metabolomics signals using hierarchical clustering analysis of the experimental samples LC-MS positive polarity. Mann Whitney *U* test was used to detect statistical significance (*p* < 0.05). **(D).** Target metabolites analyzed by LC-QqQ-MS in nasal polyps were represented in a heatmap using hierarchical clustering of the samples (represented in columns) and metabolites (in rows) showing group averages.

To assess the correct performance of the LC-MS equipment and the quality of the acquired data, samples were projected on a PCA model (Figure 2A). Clustering of the QC injections in the non-supervized plot indicated the high quality of the data, while dispersion of the samples showed the biological variability of the patients. The groups were compared using a discriminant PLS-DA analysis, obtaining no model. Thus, a Mann Whitney-*U* test was used for the selection of significant features, finding a total of 13 and 26 features from LC-MS in positive and negative ionization modes, respectively. For these significant features, we assessed that they had a coefficient of variation (CV) on QCs lower than the percentage of change between the non-allergic CRSwNP compared to the allergic CRSwNP group. Additionally, we checked that metabolites had a % of change higher than 20% or that the feature matched the complementary polarity. These features were represented using a heatmap with hierarchical clustering for each ionization mode (Figures 2B,C). As observed, the significant features from the positive ionization mode were able to define a specific metabolic signature for each group, accurately clustering all the samples. On the other hand, the significant features from the negative ionization mode were able to correctly cluster around 85% of the samples (16 out of 19).

We carried out an annotation for all the significant features. After tentative annotation and confirmation by MS/MS fragmentation experiments, we were able to annotate 14 features. Taking into account both polarities, eight metabolites had a unique annotation–comprizing sn-1 and sn-2 for lysophospholipids– (Table 2). We found increased levels of lysophosphatidylcholines (LPC 16:0, LPC 18:0 and LPC 20:4), a lysophosphatidylinositol (LPI 20:4), cortisol and bilirubin in the non-allergic CRSwNP compared to the allergic CRSwNP group.

**TABLE 2:** Pairwise comparisons showing the significant identified metabolites.

Non-allergic CRSwNP *vs* allergic CRSwNP													
N^o^	Technique	Compound	Adduct	*m/z* (Da)	Mass (Da)	RT (min)	Error (ppm)	%CV on QCs	FC in non-allergic	% Change in non-allergic	*p*-Value	*p*-BH
**1**	LC − MS −	Bilirubin	[M−H]^−^	583.2549	584.2621	32.94	2	16.9	2.03	103.28	0.041	0.049
	LC − MS +		[M + H]^+^	585.2702	584.2630	32.90	1	11.4	1.96	95.56	0.033	0.039
**2**	LC − MS +	Cortisol	[M + H]^+^	363.2171	362.2099	3.66	1	12.4	2.20	119.55	0.026	0.034
**3**	LC − MS −	LPC 16:0 sn-1	[M + FA]^-^	540.3301	495.3324	19.42	1	8.8	1.23	23.49	0.026	0.038
	LC − MS +		[M + H]+	496.3402	495.3330	19.36	1	13.5	1.27	27.17	0.036	0.042
**4**	LC − MS −	LPC 16:0 sn-2	[M + FA]^-^	540.3305	495.3324	20.20	1	6.2	1.17	16.94	0.041	0.049
	LC − MS +		[M + H]+	496.3403	495.3331	20.14	1	10.5	1.25	25.45	0.018	0.032
**5**	LC − MS −	LPC 18:0 sn-2	[M + FA]^-^	568.3623	523.3642	23.74	1	9.1	1.33	32.80	0.007	0.029
	LC − MS +		[M + H]+	524.3713	523.3641	23.66	0	12.6	1.36	36.19	0.005	0.025
**6**	LC − MS −	LPC 18:0 sn-1	[M + FA]^-^	568.3618	523.3637	24.58	1	6.2	1.23	22.55	0.041	0.049
	LC − MS +		[M + H]+	524.3719	523.3647	24.52	2	11.1	1.30	30.01	0.010	0.031
**7**	LC − MS −	LPC 20:4 sn-2	[M + Cl]^-^	578.3016	543.3324	18.59	1	10.8	1.42	42.11	0.026	0.034
	LC − MS +		[M + H]+	544.3401	543.3329	18.49	1	23.7	1.49	49.03	0.054	0.071
**8**	LC − MS −	LPI 20:4 sn-1	[M−H]^−^	619.2880	620.2952	19.59	1	6.1	1.38	38.01	0.020	0.034

LC-MS: Liquid Chromatography coupled to Mass Spectrometry; RT: retention time; ppm: part per million; FC: fold change, was calculated as average of area in Non-allergic/average of area in Allergic; % change was calculated as (FC−1) × 100; LPC: Lysophosphatidylcholine; LPI: Lysophosphatidylinositol; FA: formic acid; BH: Benjamini-Hochberg (False Discovery Rate).

Overall, specific systemic metabolic changes were defined for non-allergic and allergic CRSwNP patients.

### Targeted Metabolomic Analysis of Nasal Polyps

**FIGURE 3:**
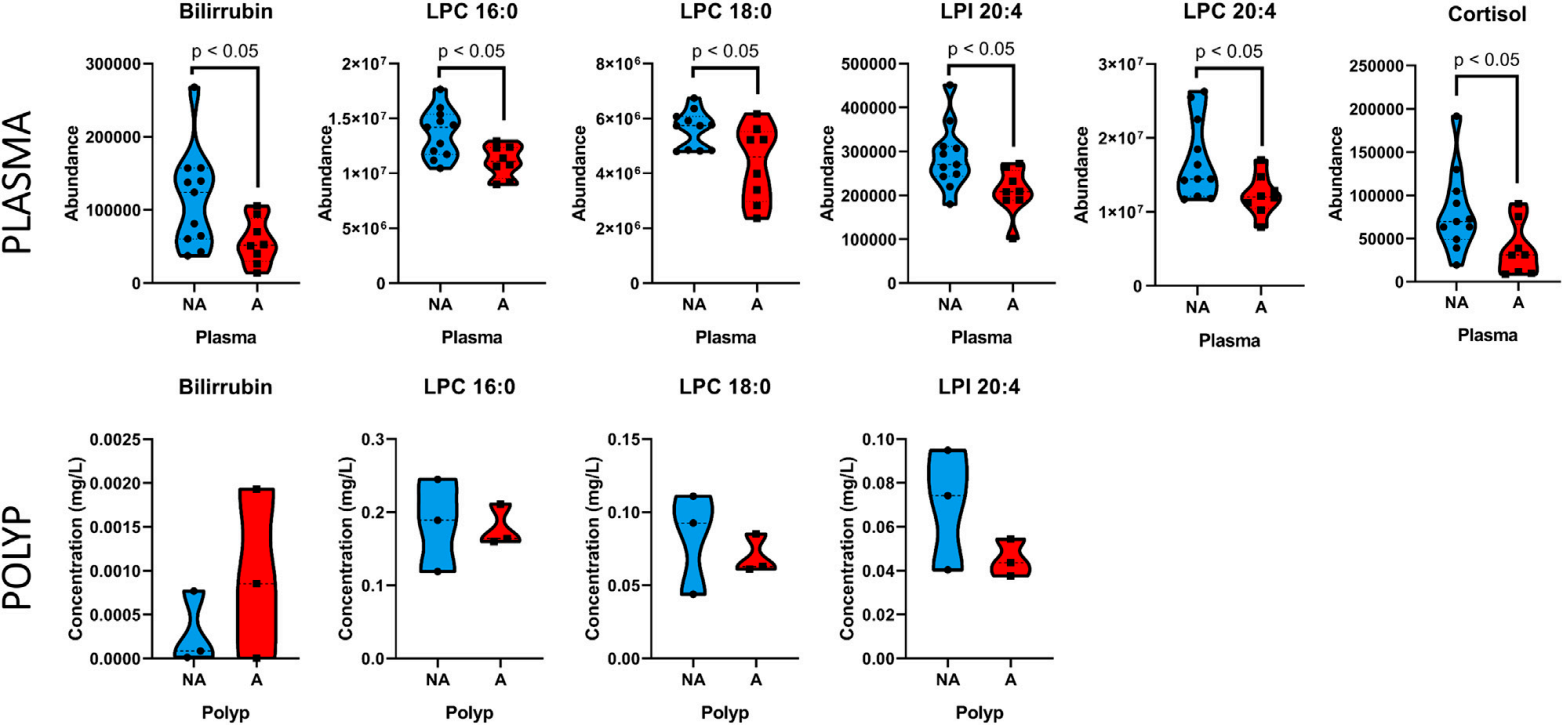
Trajectories of representative metabolites in non-allergic CRSwNP (blue), and allergic CRSwNP (red) patients in plasma and nasal polyp samples. The median is indicated by the discontinuous line within the violin plot, and each sample’s value is shown individually.

The significant metabolites found in plasma that had available commercial standards were used to test their presence in the nasal polyp. Therefore, nasal polyp samples from six CRSwNP patients (three non-allergic and three allergic) were analyzed to confirm the results found in plasma. We applied univariate statistical analysis between the two groups, obtaining no statistically significant changes in any metabolite (*p* > 0.05, Mann Whitney *U* test). However, to study the metabolite patterns, these were represented in a heatmap using the average per group (Figure 2D). We observe that, despite the low number of samples, the LPC 16:0, LPC 18:0 and LPI 20:4 followed the same increasing trend in the non-allergic CRSwNP patients as we described in plasma samples. Finally, these metabolites in plasma and in the nasal polyps from untargeted and targeted analyses were represented using trajectories in Figure 3.

### Histological Features

To better characterize the specific local features of nasal polyps and mucosa associated to allergy, we analyzed the infiltration of eosinophils, neutrophils, CD3^+^ and CD11c^+^; along with the hyperplasia of goblet cells and the abundance of collagen fibers in all of them.

**FIGURE 4:**
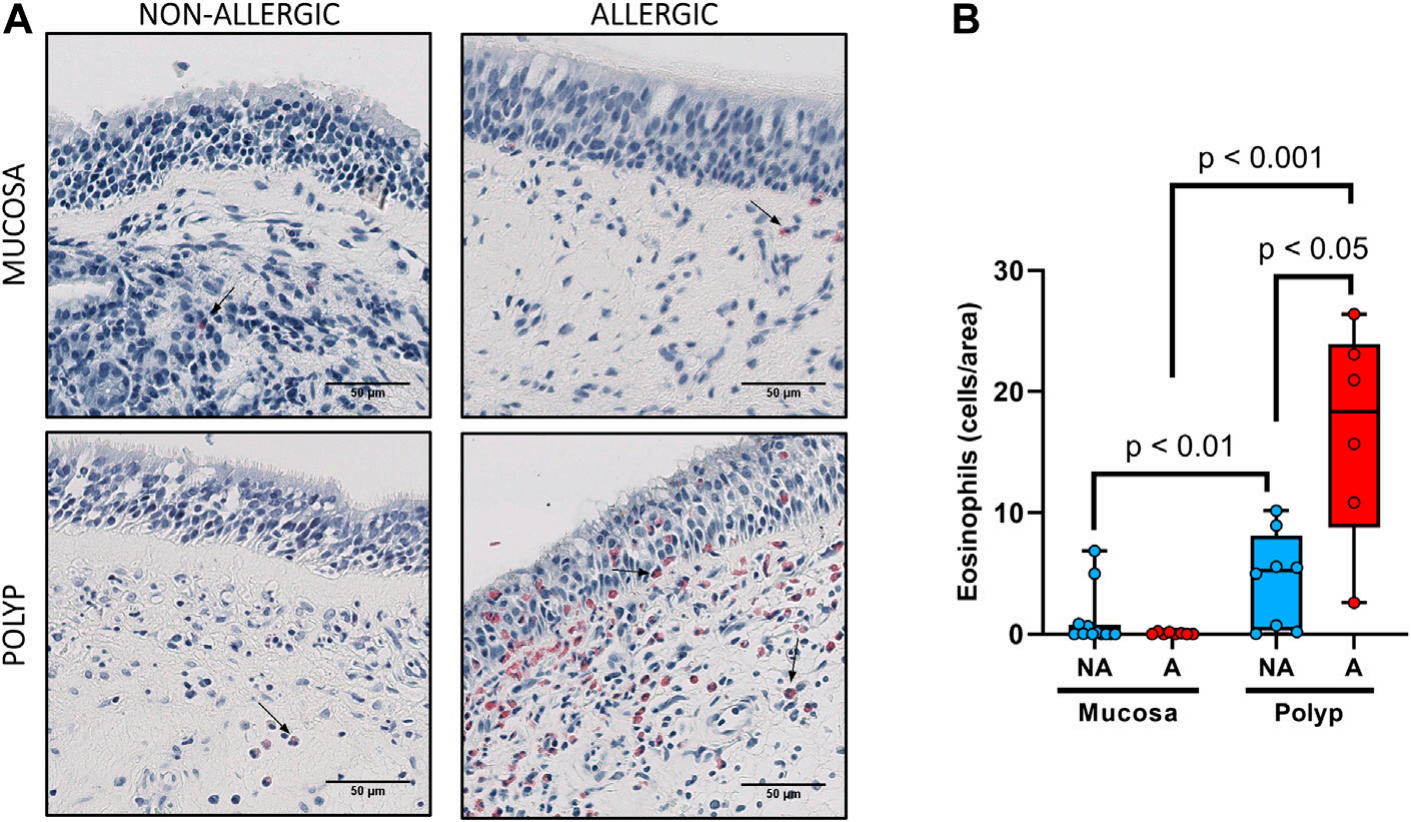
Analysis of eosinophil infiltrates in nasal polyps and nasal mucosa. **(A).** Representative images of Luna staining in nasal mucosa (*n* = 20) and nasal polyps (*n* = 14) in all experimental groups (eosinophils: black arrow →, scale bar = 50 μm). **(B).** Comparison of the number of eosinophils per area in nasal polyps and nasal mucosa samples between non-allergic CRSwNP (NA) and allergic CRSwNP (A) patients. Results are presented as mean ± SEM. Mann-Whitney *U* test was used to state significant differences.

Eosinophil quantification (Figure 4A) revealed that the number of these cells in nasal polyps was higher than in the nasal mucosa samples (9.7 ± 2.3 cells/area *vs* 0.71 ± 0.41 cells/area, *p* < 0.001). Importantly, the highest number of eosinophils was detected in nasal polyps from allergic CRSwNP patients (Figure 4B). In fact, polyps of non-allergic CRSwNP patients had significantly less eosinophils than those of allergic CRSwNP patients (16.6 ± 3.6 cells/area *vs* 4.5 ± 1.4 cells/area, *p* < 0.05).

**FIGURE 5:**
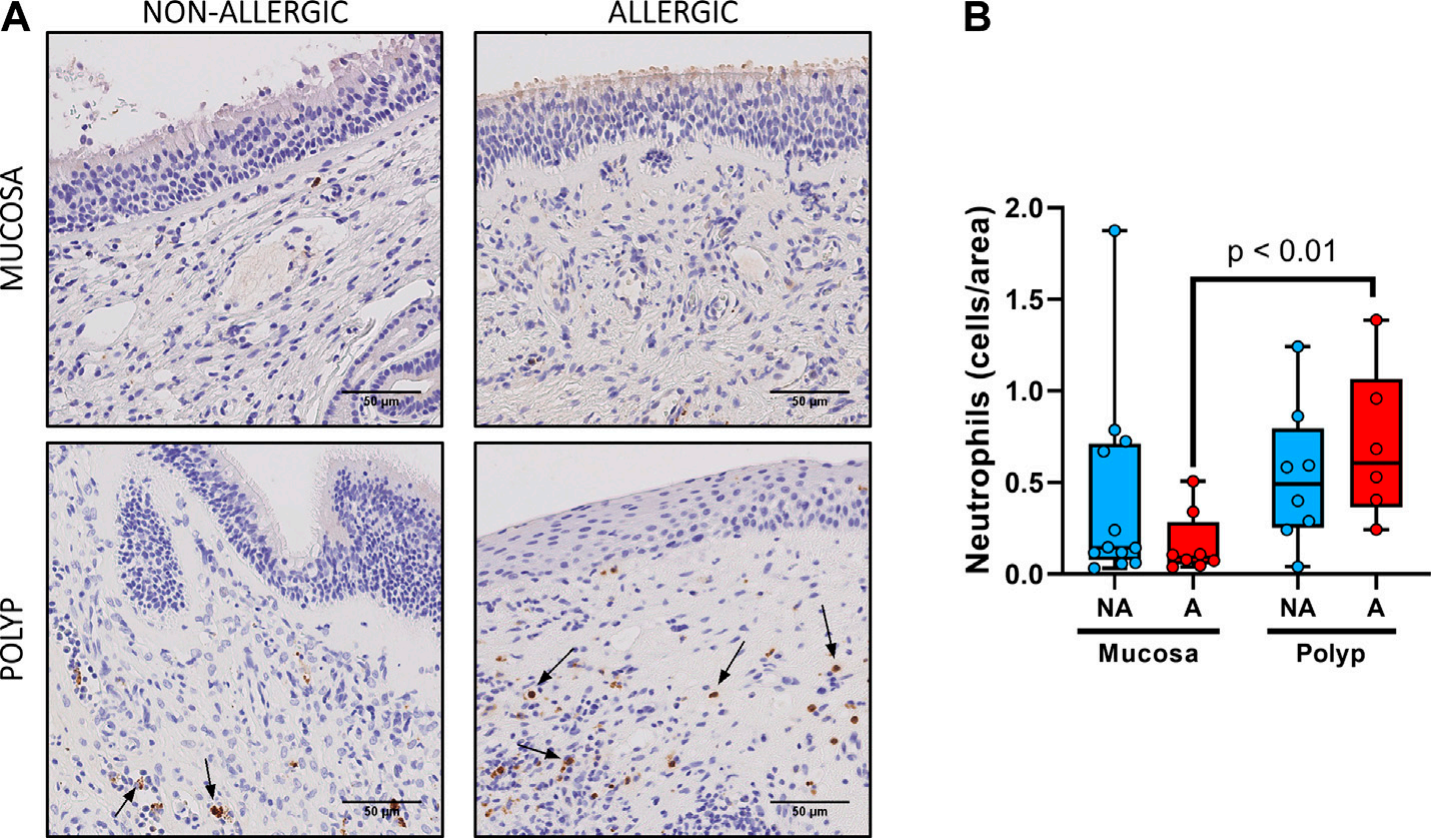
Analysis of neutrophil infiltrates in nasal polyps and nasal mucosa. **(A).** Representative images of anti-neutrophil elastase immunostaining in nasal mucosa (*n* = 20) and nasal polyps (*n* = 14) in all experimental groups (neutrophils: black arrow →, scale bar = 50 μm). **(B).** Comparison of the number of eosinophils per area in nasal polyps and nasal mucosa samples between non-allergic CRSwNP (NA) and allergic CRSwNP (A) patients. Results are presented as mean ± SEM. Mann-Whitney *U* test was used to state significant differences.

We also found more neutrophils (Figure 5A) in nasal polyps than in the nasal mucosa (0.60 ± 0.10 cells/area *vs* 0.31 ± 0.10 cells/area, *p* < 0.01). Nasal polyps of allergic CRSwNP patients had the highest number of neutrophils (Figure 5B), being significantly different to their own nasal mucosa (0.70 ± 0.17 cells/area *vs* 0.16 ± 0.060 cells/area, *p* < 0.01). However, there were no significant differences in the number of neutrophils in nasal polyps between non-allergic and allergic CRSwNP patients (*p* > 0.41).

**FIGURE 6:**
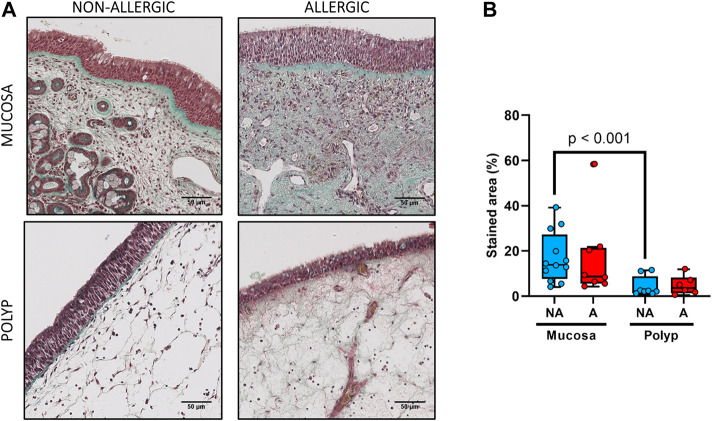
Analysis of collagen fibers deposition in nasal polyps and nasal mucosa. **(A).** Representative images of Masson-Trichrome staining in nasal mucosa (*n* = 20) and nasal polyps (*n* = 14) in all experimental groups (collagen fibers: green, cytoplasm: red, erythrocytes: gold, scale bar = 50 μm). **(B).** Comparison of the green-stained area (collagen fibers) in nasal polyps and nasal mucosa samples between non-allergic CRSwNP (NA) and allergic CRSwNP (A) patients. Results are presented as mean ± SEM. Mann-Whitney *U* test was used to state significant differences.

**FIGURE 7:**
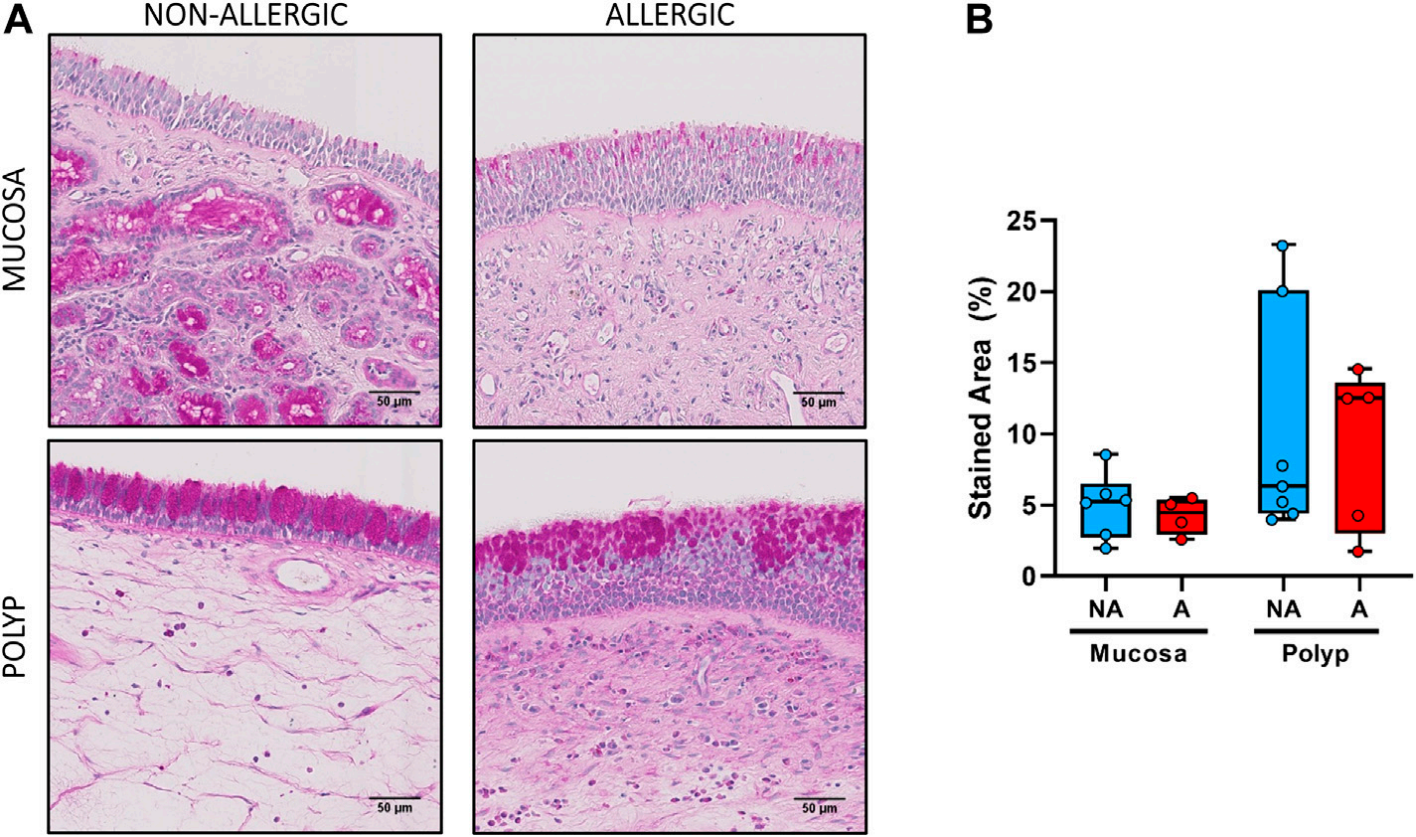
Analysis of goblet cell hyperplasia in nasal polyps and nasal mucosa. **(A).** Representative images of PAS staining in nasal mucosa (*n* = 10) and nasal polyps (*n* = 12) in all experimental groups (mucopolysaccharides: pink, nuclei: purple, scale bar = 50 μm). **B.** Comparison of the PAS-positive (pink) stained area in the epithelium of nasal polyps and nasal mucosa samples between non-allergic CRSwNP (NA) and allergic CRSwNP (A) patients. Results are presented as mean ± SEM. Mann-Whitney *U* test was used to state significant differences. Only samples with sufficient epithelial integrity were included in this analysis.

**FIGURE 8:**
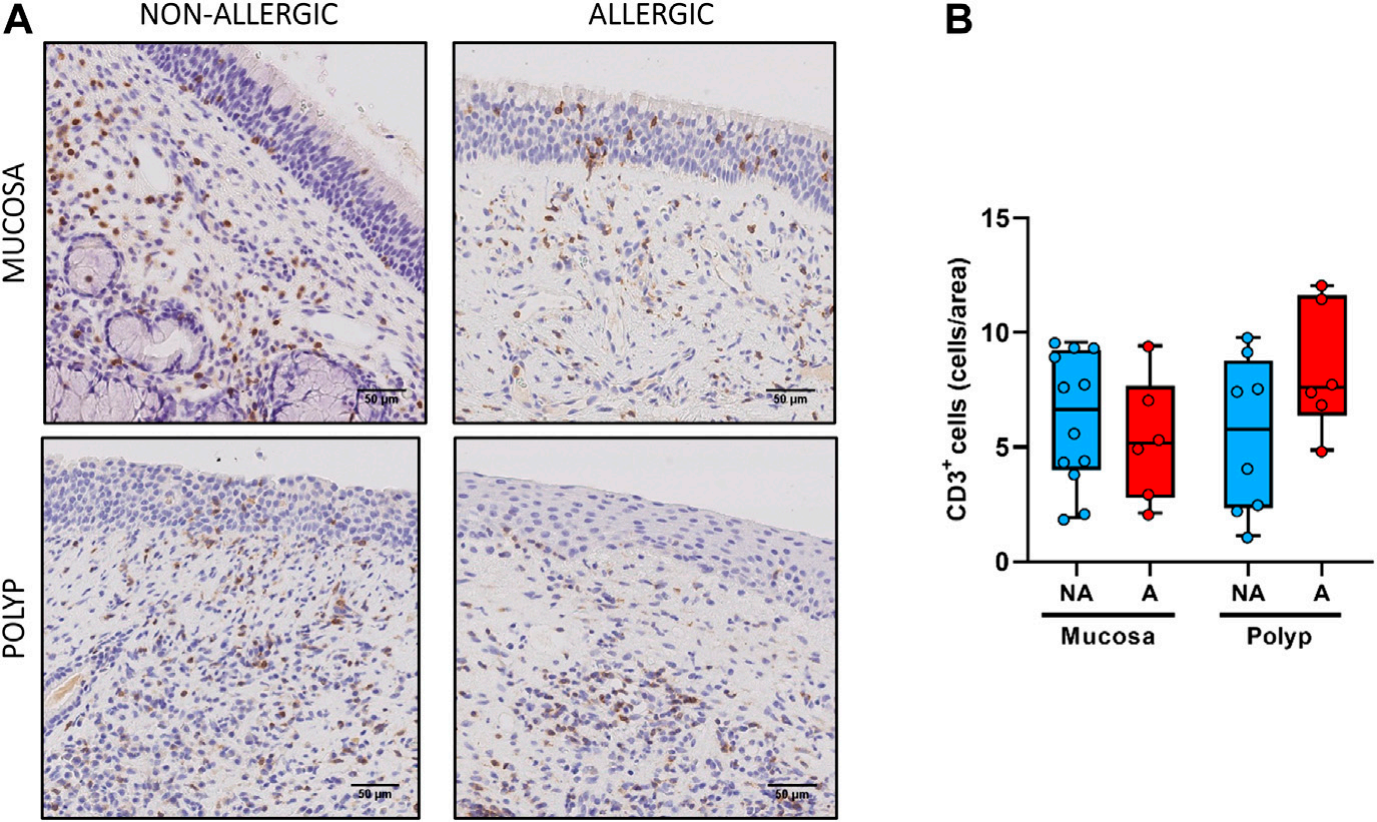
Analysis of CD3^+^ cells infiltrates in nasal polyps and nasal mucosa. **(A).** Representative images of anti-CD3 immunostaining in nasal mucosa (*n* = 18) and nasal polyps (*n* = 14) in all experimental groups (CD3^+^ cells: black arrow →, scale bar = 50 μm). **(B).** Comparison of the number of CD3^+^ cells per area in nasal polyps and nasal mucosa samples between non-allergic CRSwNP (NA) and allergic CRSwNP (A) patients. Results are presented as mean ± SEM. Mann-Whitney *U* test was used to state significant differences.

**FIGURE 9:**
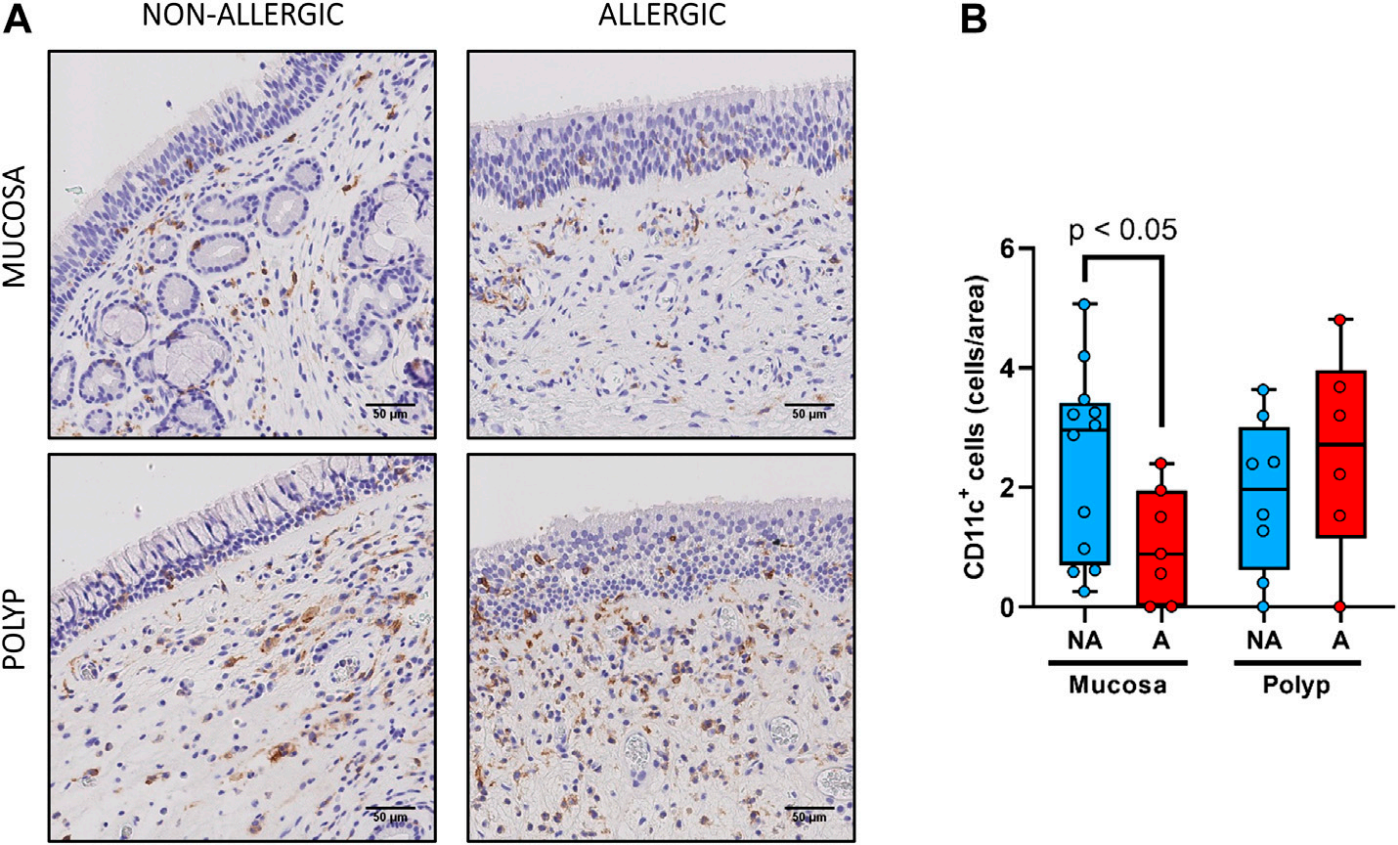
Analysis of CD11c^+^ cells infiltrates in nasal polyps and nasal mucosa. **(A).** Representative images of anti-CD11c immunostaining in nasal mucosa (*n* = 19) and nasal polyps (*n* = 14) in all experimental groups (CD11c^+^ cells: black arrow →, scale bar = 50 μm). **(B).** Comparison of the number of CD11c^+^ cells per area in nasal polyps and nasal mucosa samples between non-allergic CRSwNP (NA) and allergic CRSwNP (A) patients. Results are presented as mean ± SEM. Mann-Whitney *U* test was used to state significant differences.

No statistical differences were observed between the nasal polyps of non-allergic CRSwNP and those of allergic CRSwNP regarding collagen deposition (Figure 6, *p >* 0.66), goblet cell hyperplasia (Figure 7, *p <* 0.87), infiltration of CD3^+^ (Figure 8, *p <* 0.22) and CD11c^+^ cells (Figure 9, *p >* 0.41).

However, more collagen deposition was observed in the nasal mucosa compared to the nasal polyps in the non-allergic CRSwNP (*p* < 0.001). Moreover, the same trend was found for the allergic CRSwNP patients, although the difference was not significant (*p* > 0.059) (Figure 6B).

There also seemed to be a higher area stained with PAS in the epithelium of nasal polyps compared to the nasal mucosa for both allergic and non-allergic CRSwNP groups (Figure 7). Although the difference was not significant (*p >* 0.29 for non-allergic CRSwNP and *p >* 0.41 for allergic CRSwNP patients), this fact suggests the presence of goblet cell hyperplasia.

Finally, for the infiltration of CD3^+^ and CD11c^+^ cells (Figures 8,9, respectively), no statistical differences were obtained between nasal mucosa and nasal polyp for both allergic and non-allergic CRSwNP groups.

In summary, we found that nasal polyps present a higher immune cell infiltration (eosinophils and neutrophils) than nasal mucosa for both allergic and non-allergic CRSwNP patients. Moreover, allergic CRSwNP patients were characterized by eosinophilia in their nasal polyps compared to non-allergic CRSwNP patients. In the case of non-allergic CRSwNP patients, more collagen deposition in their nasal mucosa than in their nasal polyp was observed.

## Discussion

Nasal polyps are growths of inflamed nasal tissue that have been well-known for a long time. However, the molecular mechanisms involved in the development of nasal polyps remain unclear. Additionally, even though allergy and CRSwNP have been traditionally linked, whether there is a connection between allergy and the development of nasal polyps or not is yet to be described. Here, we perform an original experimental design, aiming to elucidate systemic metabolic differences in CRSwNP patients with and without allergy.

From our patient cohort, 40% (9 out of 22) of the patients were allergic, close to the levels in average population which is around 30% (Bauchau and Durham, 2004; Bousquet et al., 2008).

A metabolic fingerprint in plasma of patients with CRSwNP was obtained. Non-allergic CRSwNP patients displayed an increase of LPCs (LPC 16:0, LPC 18:0, LPC 20:4) together with LPI 20:4, compared to allergic CRSwNP. These LPCs have been previously associated with systemic inflammation (Chiurchiù et al., 2018; Obeso et al., 2018). Moreover, they have been described in asthma (Ried et al., 2013; Comhair et al., 2015; Villaseñor et al., 2017) as arachidonic acid (AA) precursors. The free fatty acids from the LPCs are released after the action of lipase A2 to synthesize inflammatory mediators, that participate as precursors in the AA pathway (Balgoma et al., 2010; Arita, 2016; Bennett and Gilroy, 2016). Interestingly, LPI 20:4, which is one of the more abundant LPIs in plasma, has been related to a potent pro-inflammatory signaling in intestinal bowel disease and colorectal cancer in animal models (Grill et al., 2019) and in type 2 diabetes (Lu et al., 2016). Overall, the LPI 20:4 metabolite seems to be involved in the inflammatory response.

On the other hand, there is growing evidence that bilirubin exerts potent anti-inflammatory effects. Bilirubin is able to suppress inflammatory responses by preventing the migration of leukocytes into target tissues through the disruption of vascular cell adhesion molecule-1 (VCAM-1)-dependent cell signaling. In a previous study, bilirubin was shown to alleviate colitis (Vogel and Zucker, 2016). Additionally, nanoparticles containing bilirubin were used for the treatment of allergic lung inflammation disease in a mouse model obtaining amelioration of the disease (Kim et al., 2017). Therefore, bilirubin has been demonstrated that has anti-oxidative, anti-inflammatory and immunosuppressive functions in various diseases such as inflammatory bowel disease, cardiovascular disease, autoimmune disorders, cancer and type 2 diabetes mellitus (Peng et al., 2017). Mildly elevation of this metabolite is associated with better prognosis; thus, the decrease we observe in allergic CRSwNP patients might be related with a worse outcome. As bilirubin can be measured in a laboratory standard test, this metabolite can be compared in future studies in these patients.

Finally, cortisol has been described as a hormone which acts suppressing early inflammatory responses; however, when cortisol is not enough to control inflammation, it prepares immune cells for major subsequent inflammatory episodes (Yeager et al., 2018). Interestingly, circadian rhythms of salivary cortisol were found to be disrupted in patients with extensive nasal polyposis compared to controls (Fidan et al., 2013). The authors suggest that a therapy with cortisol-based drugs might be useful in the treatment of CRSwNP (Fidan et al., 2013). In this line, intrapolyp steroid injections appear to be effective and safe for the treatment of nasal polyps (Kırıs et al., 2016). Additionally, it has been shown that polyp-derived epithelial cells produce cortisol, which may be involved in the anti-inflammatory response established when patients receive treatment with glucocorticoid for nasal polyps (Kook et al., 2015). The elevation of this metabolite in the non-allergic CRSwNP patients might point to a specific pathway to cope with inflammation. Additionally, this suggests that, in allergic CRSwNP patients, the cortisol acts by recruiting immune cells instead of solving the inflammation by itself.

Our results demonstrate that allergy produces metabolic changes in plasma in patients with CRSwNP. The increase of LPC 16:0, LPC 18:0, LPC 20:4, LPI 20:4, cortisol and bilirubin metabolites in the plasma of non-allergic CRSwNP patients points toward a systemic inflammatory response in the absence of allergy. These metabolites might participate in the development of a characteristic tissue environment, responsible for the differences observed at the histological and inflammatory infiltration levels. Therefore, we hypothesized that they might be also altered in the nasal polyp in the same way.

Consequently, we implemented a novel target methodology to analyze these significant metabolites from plasma that were commercially available in the nasal polyp, obtaining good analytical parameters. We observed that the trends of the LPC 16:0, LPC 18:0 and LPI 20:4 metabolites are similar in plasma and nasal polyps samples. However, further studies are needed, including a higher number of tissue samples to validate these results.

Together, the metabolomic results demonstrate that allergy induces specific metabolic changes in CRSwNP patients. These are LPC 16:0, LPC 18:0, LPC 20:4, LPI 20:4, cortisol and bilirubin. Most of these metabolites were measured in nasal polyp, a biological sample that has not been previously analyzed by metabolomics. We showed that LPC 16:0, LPC 18:0 and LPI 20:4 follow the same trend in the nasal polyp than in plasma. This novel methodological procedure could be used in future studies to better understand the local metabolomic environment of nasal polyps.

Histological characteristics in the nasal polyp and their nasal mucosa of these patients was also analyzed. Tissue eosinophils have been described both in nasal polyps and in allergic pathologies. In fact, treatment with anti-IL5 (mepolizumab) has shown a reduction in both nasal polyp and blood eosinophils, and a significant improvement in CRSwNP patients, resulting in better prognosis (Bachert et al., 2017). Eosinophils have been also measured locally and systemically. Therefore, a high number of tissue eosinophils and/or their proteins in nasal polyps have been related to more severe and maintained symptoms (Sun et al., 2017) and recurrence (Tos et al., 2010; Van Zele et al., 2014; Yang et al., 2018). High numbers of eosinophils in plasma correlate with higher allergy incidence and worse symptomatology as well (Erbek et al., 2007). Thus, it seems clear that eosinophils might play a role in the development and prognosis of allergic CRSwNP.

On the other hand, neutrophil quantification showed a similar distribution pattern between non-allergic CRSwNP and allergic CRSwNP patients, which was independent of the presence or absence of eosinophils. Although there are studies describing lower numbers of neutrophils in eosinophilic CRSwNP compared to the non-eosinophilic polyps (Schleimer, 2017); other authors (Pothoven et al., 2017; Kong and Kim, 2018) have recently reported similar results, linking the role of neutrophils in this disease to their expression of oncostatin M (OSM), a cytokine that has been found elevated in CRSwNP and that induces barrier dysfunction.

Additionally, a lower collagen deposition in nasal polyps compared to nasal mucosa for both allergic and non-allergic CRSwNP patients, suggest that the polyps of the study are edematous, rather than a fibrous (Brescia et al., 2021).

In brief, immune cell infiltration analysis revealed differential features between nasal polyp and nasal mucosa and suggest that a maintained allergy would enhances the inflammatory response mediated by eosinophils in nasal polyps but, surprisingly, not in nasal mucosa for allergic CRSwNP patients.

Separately, according to Brescia *et al* (Brescia et al., 2021), CRS appears to be a very heterogeneous inflammatory condition, with various emerging endotypes. Molecular and cellular screening together with clinical phenotyping in CRSwNP could be useful to define endotypes, and thus clarify the inflammatory mechanisms and allow the establishment of a more precise treatment. However, because of the aim of this exploratory study was to use allergy as the main classifying criterion, we are not able to describe our data in terms of CRS endotypes. This limitation should be addressed in the future including a more complete immunological, histopathological and clinical phenotyping approach, what will lead to an improvement in the endotyping capability, as it has been reported (Brescia et al., 2017; Kim and Cho, 2017; Bachert et al., 2018). In this sense, metabolomics could offer help in both 1) the endotyping of patients with CRSwNP, along with cellular and other molecular analysis; and 2) the understanding of the molecular mechanisms that underlay the endotypes.

Our findings suggest that the two phenotypes (non-allergic CRSwNP and allergic CRSwNP), although sharing histological features such as cellular infiltration, have distinctive metabolomic fingerprints and eosinophilia, which point toward different mechanisms of formation.

Previous studies from our group have followed this metabolomics approach in other respiratory allergy models and have found alterations in the AA pathway, as we report in this manuscript (Obeso et al., 2018; Barker-Tejeda et al., 2020). Although, surprisingly, this route was downregulated in allergic CRSwNP patients, an increased recruitment of eosinophils in the nasal polyp was observed. Therefore, we hypothesized that the development of the phenotype CRSwNP without allergy requires a great underlying uncontrolled systemic inflammation, which is different in the allergic phenotype. Thus, eosinophils seem to be accountant for the inflammation leading to CRSwNP development in the allergic phenotype, while non-allergic CRSwNP phenotype would be characterized by higher levels of AA precursors and other inflammatory mediators needed to develop the nasal polyp. The reason of the increase in these biological pathways is, however, yet to be defined.

This is an exploratory study, where the design is innovative and aimed to understand the effect of respiratory allergy in the development of CRSwNP; however, it has some limitations. Although the sample size was small, the samples (nasal polyps, nasal mucosa and plasma) were extensively characterized by metabolomics and histology. Moreover, despite the metabolic alterations that were observed between these two groups of patients not being able to generate a discriminant model (i.e. PLS-DA) capable of predicting new samples, the findings are promising in this field and would shed light on the mechanism by which patients without allergy develop CRSwNP. Therefore, in further studies, the validation of these results in a bigger cohort is needed.

Overall, CRSwNP is a pathology of high level of clinic-pathological complexity where the collection of biopsies is not an easy task. Although the role of histological study of biopsies is a complementary approach for the endotyping of nasal polyps, the inclusion of a metabolomic analysis has allowed us to identify biological processes associated with the allergic or non-allergic phenotypes and, therefore, could be helpful in the design of novel, less invasive treatments for these patients.

## Conclusion

Our results demonstrate that patients with CRSwNP with and without allergy display systemic metabolic changes. Surprisingly, these metabolites (LPC 16:0, LPC 18:0, LPC 20:4, LPI 20:4, cortisol and bilirubin), which are associated with inflammation, appear to be increased in the absence of allergy, suggesting that non-allergic CRSwNP patients develop the nasal polyp after a sustained systemic inflammation. We have developed a new method for the analysis of nasal polyps using targeted metabolomics. With this, we spotted the same trends of LPC 16:0, LPC 18:0 and LPI 20:4 that were observed in plasma. The increased numbers of eosinophils in the nasal polyp of allergic CRSwNP patients hints that nasal polyps might develop by a local immune effector cell recruitment that ends in tissue remodeling. Finally, this is an exploratory study, where the significant metabolites were obtained by a semi-quantitative comparison between the groups. Thus, interpretation of these results should be made taking into account these limitations. Further validation studies in new cohort of samples using targeted quantitative methods must be carried out.

## Data Availability

The datasets presented in this study can be found in online repositories. The names of the repository/repositories and accession number(s) can be found below: https://www.metabolomicsworkbench.org/, ST001733;https://www.metabolomicsworkbench.org/, ST001734.
