# Supplementary material for: Understanding Systemic and Local Inflammation Induced by Nasal Polyposis: Role of the Allergic Phenotype
Source: Front Mol Biosci. 2021 May 14;8:662792. doi: 10.3389/fmolb.2021.662792 (PMC8160224; doi:10.3389/fmolb.2021.662792)
Supplement: Supplementary file 1 [file DataSheet1.docx]

**Supplementary Information**

**Understanding systemic and local inflammation induced by nasal polyposis: role of the allergic phenotype.**

María Isabel Delgado-Dolset^1^, David Obeso^1,2^, Javier Sánchez-Solares^1^, Leticia Mera-Berriatua^1^, Paloma Fernández^1^, Coral Barbas^2^, Miguel Fresnillo^3^, Tomás Chivato^1,4^, Domingo Barber^1^, María M Escribese^1,5,†^, Alma Villaseñor^1,†,*^

^1^Institute of Applied Molecular Medicine, Department of Basic Medical Sciences, Faculty of Medicine, San Pablo CEU Universities, Madrid, Spain

^2^Centre for Metabolomics and Bioanalysis (CEMBIO), Department of Chemistry and Biochemistry, Facultad de Farmacia, Universidad San Pablo-CEU, CEU Universities, Urbanización Montepríncipe, 28660 Boadilla del Monte. Madrid, Spain.

^3^Otorhinolaringology Service, HM Montepríncipe Hospital, Boadilla del Monte, Madrid, Spain

^4^Department of Clinic Medical Sciences, Faculty of Medicine, San Pablo CEU University, Madrid, Spain

^5^Department of Basic Medical Sciences, Faculty of Medicine, San Pablo CEU University, Madrid, Spain

†These authors share last authorship

**Material and Methods**

*Detailed description of sample preparation, instrumental description, data pre-treatment and Quality Assurance for metabolomics analyses.*

Plasma proteins were removed by adding 300 µL of cold (-20 ℃) methanol:ethanol (1:1) to 100 µL of sample. Samples were then vortex-mixed and stored on ice for 5 min. Supernatant containing the metabolites was separated from the pellet by centrifugation (16,000 × g for 20 min at 4 ℃), then put into a LC vial for analysis.

Quality control sample (QC) was prepared by pooling equal volumes of plasma from each sample. QC followed the same procedure applied for the experimental samples and was analysed throughout the run to provide a measurement of system stability, performance and reproducibility. All samples were randomised before metabolite extraction and for the corresponding analytical run.

Analysis was performed on an Agilent HPLC system (1200 series, Agilent Technologies, Waldbronn, Germany) equipped with a degasser, two binary pumps, and a thermostated auto sampler coupled to a quadrupole-time of flight analyser (Q-TOF), LC-MS 6520 system (Agilent Technologies, Waldbronn, Germany). A volume of 10 μL of sample were injected into a Discovery HS C18 column (2.1 mm × 150 mm, 3.0 μm; Supelco, Sigma Aldrich, Germany), with a guard column Discovery® HS C18 (2 cm × 2.1 mm, 3 μm; Supelco, Sigma Aldrich, Germany), both maintained at 40 ℃. The flow rate was set at 0.6 mL/min. The elution gradient involved a mobile phase consisting of: (A) 0.1% formic acid (FA) in water, and (B) 0.1% FA in acetonitrile. Initial conditions were set at 25% phase B, which increased to 95% phase B in 35 min; then, it was re-equilibrated for 1 min and finally held for 9 min in the initial conditions. The electrospray source ionization (ESI) data were acquired in both positive and negative ion modes, respectively. The capillary voltage was set at 3,500 for ESI (+) and 4,000 V for ESI (−). The drying gas flow rate was 10.5 L/min at 330 ℃ and gas nebulizer at 52 psi; fragmentor voltage was 175 V; skimmer and octopole radio frequency voltage (OCT RF Vpp) were set to 65 and 750 V. Data were collected in the centroid mode at a scan rate of 1.2 spectra per second. Mass spectrometry detection was performed in full scan from 100 to 1200 *m/z* for both positive and negative ESI modes. The reference *m/z* ions were purine (121.0508) and HP-0921 (922.0097) for ESI (+), whereas TFA NH4 (119.0363) and HP-0921 (966.0007) for ESI (−). These masses were continuously infused into the system to allow constant mass correction. Samples were analysed in separate runs.

Acquired data were cleaned of background noises and unrelated ions using MassHunter Profinder (B.06.00, Agilent Technologies) software. “Molecular feature extraction” and “Find by ion” algorithms were applied to reduce the size and complexity of data, and to improve the reliability in finding the features. 1654 and 698 chemical signals for LC-MS positive and negative ionization modes were obtained, respectively. Then, data was filtered, and only those features detected in >50% in QCs and with a Relative Standard Deviation (RSD) <30% in Qcs were kept, resulting in 535 for LC-MS positive and 429 for negative ionization mode.

Multivariate analysis was performed using MetaboAnalyst v5.0. We used Principal Component Analysis (PCA) with a logarithmic transformation and centre scaling to observe data quality. Statistical analyses were performed using Matlab R2015a (Mathworks) software. Differences among the pairwise comparison were tested using the non-parametric Mann Whitney U test, with statistical significance set at 95% (p<0.05) with a Benjamini-Hochberg (also known as False Discovery Rate, FDR). The MetaboAnalyst online tool was also used for heatmaps and hierarchical clustering.

Compound annotation for LC-MS was carried out for statistically significant features in two steps. First, a putative identification was performed in all the significant features by searching the accurate masses against the online available databases such as KEGG, METLIN, LipidMaps and HMDB using the CEU Mass Mediator tool [1-3]. Then, identity was confirmed through LC-MS/MS experiments by using a QTOF (model 6520, Agilent). Experiments were repeated in identical chromatographic conditions as the primary analyses. Ions were targeted using the narrow *m/z* window (1.3 Da) and 20 eV of energy for fragmentation on the quadrupole. Comparison of the structure proposed against the obtained fragments led to the confirmation of the identity.

1. Gil-de-la-Fuente A, Godzien J, Saugar S, Garcia-Carmona R, Badran H, Wishart DS, et al. CEU Mass Mediator 3.0: A Metabolite Annotation Tool. J Proteome Res. 2019;18(2):797–802.

2. Godzien J, Alonso-Herranz V, Barbas C, Armitage EG. Controlling the quality of metabolomics data: new strategies to get the best out of the QC sample. Metabolomics. 2015;11(3):518–28.

3. Dudzik D, Barbas-Bernardos C, García A, Barbas C. Quality assurance procedures for mass spectrometry untargeted metabolomics. a review. Vol. 147, Journal of Pharmaceutical and Biomedical Analysis. Elsevier B.V.; 2018. 149–73.
